# Assessment of Corneal Sensation, Innervation and Retinal Nerve Fiber Layer in Patients Treated with Multiple Intravitreal Ranibizumab Injections

**DOI:** 10.1371/journal.pone.0170271

**Published:** 2017-01-13

**Authors:** Gulfidan Bitirgen, Selman Belviranli, Rayaz A. Malik, Hurkan Kerimoglu, Gunhal Satirtav, Nazmi Zengin

**Affiliations:** 1 Department of Ophthalmology, Necmettin Erbakan University Meram Faculty of Medicine, Konya, Turkey; 2 Weill Cornell Medicine-Qatar, Education City, Doha, Qatar & Central Manchester University Teaching Hospitals Foundation Trust and Division of Cardiovascular Sciences, University of Manchester, Manchester, United Kingdom; Charite Universitatsmedizin Berlin, GERMANY

## Abstract

**Purpose:**

To evaluate the effects of repeated intravitreal ranibizumab injections on corneal sensitivity, corneal sub-basal nerve plexus (SBNP) and peripapillary retinal nerve fiber layer (RNFL) thickness in patients with neovascular age-related macular degeneration (AMD).

**Methods:**

Sixty-six eyes of 33 patients who had received unilateral repeated intravitreal ranibizumab injections (0.5 mg/0.05 ml) for the treatment of AMD and 25 eyes of 25 healthy subjects were included in the study. Central corneal sensation was measured using the contact Cochet-Bonnet esthesiometer. The laser scanning in vivo corneal confocal microscope was used to determine corneal SBNP parameters. The peripapillary RNFL thickness was assessed with spectral-domain optical coherence tomography. Data obtained from the ranibizumab-injected eyes were compared with those of the fellow non-treated eyes and the eyes of the healthy control subjects.

**Results:**

The mean number of ranibizumab injections per eye was 8.9±5.0 (range 3–20). There were no statistically significant differences in the central corneal sensitivity threshold and corneal SBNP parameters between the ranibizumab-injected eyes and the fellow untreated eyes or between those with neovascular AMD and the healthy control group (P>0.05 for all). The average peripapillary RNFL thickness of the treated eyes did not differ significantly to the fellow eyes (P = 0.237), and the eyes of healthy control subjects (P = 0.918). There were no significant correlations between the number of ranibizumab injections and any of the study parameters.

**Conclusions:**

Multiple intravitreal injections of ranibizumab seem to have no harmful effects on corneal sensitivity, innervation and peripapillary RNFL thickness in patients with AMD.

## Introduction

Age-related macular degeneration (AMD) is the leading cause of irreversible blindness among elderly people in developed countries [1]. Neovascular or exudative AMD, characterized by new formation of choroidal vessels into the sub-retinal space, is responsible for almost 90% of severe vision loss due to AMD [2]. Although the age-related changes that stimulate pathologic neovascularization are not completely understood, vascular endothelial growth factor A (VEGF-A), which is a key regulator of angiogenesis and vascular permeability, has been implicated as an important factor in the pathogenesis of AMD [3,4].

Ranibizumab, a recombinant humanized monoclonal antibody antigen-binding fragment that neutralizes all known active forms of VEGF-A, has widely been used via intravitreal injection for the treatment of neovascular AMD [5]. However, the effect of VEGF is not limited to angiogenesis and vascular permeability. Reassuringly a recent study has shown no effect on corneal endothelial cell density or morphology after intravitreal bevacizumab or ranibizumab in patients with diabetic macular edema [6]. However, experimental studies have documented that VEGF is expressed by neurons mediating neuroprotective and neurotrophic activities [7–9], and neutralization of VEGF, using anti-VEGF antibodies impairs regeneration of the corneal sub-basal nerve plexus (SBNP) in injured mice corneas [10]. Repeated intravitreal injections of ranibizumab might therefore induce a detrimental effect on the corneal SBNP and retinal nerve fiber layer (RNFL). Several studies have investigated the effects of anti-VEGF on RNFL, reporting either decreased, increased or unchanged RNFL thickness in the eye treated with intravitreal anti-VEGF agents [11–15]. To our knowledge, there are no data in the current literature regarding the effects of intravitreal anti-VEGF agents on the human corneal SBNP.

In this study, we evaluated the effects of repeated intravitreal ranibizumab injections on corneal sensitivity, corneal SBNP morphology and peripapillary RNFL thickness in patients with neovascular AMD.

## Materials and Methods

Thirty-three patients (16 male, 17 female) with unilateral neovascular AMD who had received at least three injections of intravitreal ranibizumab (Lucentis; Novartis Pharma AG, Basel, Switzerland) and 25 healthy control subjects (14 male, 11 female) were enrolled in this cross-sectional study undertaken at a tertiary referral center. The study design fulfilled the tenets of the Declaration of Helsinki and was approved by the Clinical Research Ethics Committee of the Necmettin Erbakan University. Written informed consent was obtained from all patients after a detailed explanation of the nature and possible consequences of the study.

Medical records of all patients were reviewed for age, gender, pre-existing glaucoma, use of anti-glaucomatous medications and clinical examination findings. The total number of intravitreal injections and the time elapsed from the last injection were also recorded. The patients had received initially three monthly intravitreal ranibizumab (0.5 mg/0.05 ml) injections. Afterward, re-injections had been performed according to an “as needed” protocol based on the PrONTO trial [16]. None of the patients experienced systemic or ocular complications including intraocular inflammation, endophthalmitis, or thromboembolic events. Patients were excluded if they had a history of glaucoma, elevated intraocular pressure (IOP) during follow-up, trauma or ophthalmic surgery, any corneal pathology and coexisting retinal or neuro-ophthalmologic diseases. Patients with systemic diseases other than controlled hypertension were also considered ineligible.

All patients underwent a complete ophthalmologic evaluation, including anterior segment biomicroscopy, IOP assessment and fundus examination. The patients with neovascular AMD were included if the fellow eye was without AMD findings or just early dry AMD. Data obtained from the ranibizumab-injected eyes were compared with those of the fellow eyes. Since the intravitreal administration of ranibizumab may affect the fellow eyes of the patients via the systemic circulation, 25 eyes of 25 healthy subjects were also included.

Central corneal sensation was measured before the IOP assessment, using a contact nylon thread esthesiometer (Luneau 12/100 mm Cochet-Bonnet; Luneau, Prunay le Gillon, France). The nylon filament, which mechanically stimulates corneal nerves, was applied with a low pressure perpendicularly to the center of the cornea. Starting from 6 cm, the filament length was progressively reduced in 5-mm steps until the first response occurred. The longest filament length (cm) resulting in a positive response was verified twice and recorded as the indicator of the central corneal sensitivity [17].

The laser scanning in vivo corneal confocal microscopy (IVCCM) was performed on all subjects using the Rostock Corneal Module/Heidelberg Retina Tomograph lll (RCM/HRT lll; Heidelberg Engineering GmBH, Dossenheim, Germany). The full thickness of the central cornea was scanned by using the device’s “section” mode. The total duration of IVCCM examination was approximately 2 minutes per eye, and none of the subjects experienced any visual symptoms or corneal complications as a result of examination. The best-focused SBNP images were considered for the analysis. Automatic CCMetrics software, ver. 2.0 (University of Manchester, UK) was used for the quantitative analysis of the nerve fibers [18]. Three parameters were quantified: corneal nerve fiber density (NFD), the total number of major nerves per square millimeter; nerve fiber length (NFL), the total length of all nerve fibers and branches (millimeters per square millimeter); and nerve branch density (NBD), the number of branches emanating from major nerve trunks per square millimeter [19]. All of the image analyses were performed by a single masked observer.

The peripapillary RNFL thickness measurements were obtained using a spectral-domain optical coherence tomography (OCT) (Heidelberg Engineering, Heidelberg, Germany). The OCT scanning circle (size: 3.4 mm) was manually positioned at the center of the optic disc and the average peripapillary RNFL thickness (μm) was recorded.

Statistical analysis was performed using SPSS ver. 17.0 (Chicago, IL, USA) software. Basic descriptive statistics were calculated on all the data gathered and are reported as the mean±SD or n (%), as appropriate. The Pearson *χ*^2^ test was used to compare the categorical parameters. Normal distribution of continuous variables was confirmed with the Shapiro-Wilk test. Paired samples t-test or Wilcoxon signed ranks tets was used to compare the parameters between the ranibizumab-injected eyes and fellow eyes. Independent samples t-test or Mann-Whitney test was used to compare the parameters between the ranibizumab-injected eyes and the eyes of healthy control subjects. The correlations between the number of injections and corneal sensitivity threshold, peripapillary RNFL thickness and corneal SBNP parameters were measured by using Pearson’s correlation coefficient. For all evaluations, a P value of less than 0.05 was considered statistically significant.

## Results

The mean ages of the patients and control group were 67.91±7.29 years (range 55–81 years) and 66.24±7.24 years (range 56–85 years), respectively. There were no statistically significant differences between patients with AMD and healthy controls for age (P = 0.391) and gender (P = 0.571). No significant differences were observed in central corneal sensitivity threshold, corneal SBNP parameters and peripapillary RNFL thickness between male and female genders in both the AMD and control groups (P>0.05 for all). The mean number of injections was 8.9±5.0 (range 3–20) and the mean time elapsed from the last intravitreal injection treatment was 2.7±1.7 months (range 1–6 months). The mean IOP of eyes receiving ranibizumab injections (14.79±2.58 mmHg) did not differ significantly from those of the fellow eyes (14.73±2.75 mmHg) and control eyes (13.56±2.66 mmHg) (P = 0.837 and P = 0.082, respectively).

**Fig 1:**
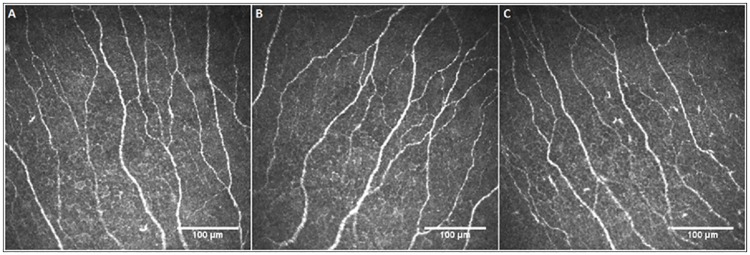
In vivo corneal confocal microscopic images of the central cornea showing the sub-basal nerve plexus in the ranibizumab-injected eye (A) and fellow eye (B) of a patient with neovascular AMD and the eye of a healthy control subject (C).

Table 1 shows the comparison of central corneal sensitivity threshold, corneal SBNP parameters and peripapillary RNFL thickness among ranibizumab-injected eyes and fellow eyes of the patients with AMD and the healthy control group. Representative IVCCM images of the central cornea showing the SBNP in a patient with neovascular AMD and a healthy control subject are given in Fig 1. There were no statistically significant differences between the ranibizumab-injected eyes and fellow eyes, as well as the ranibizumab-injected eyes and control eyes (P>0.05 for all). In order to detect any early changes on corneal nerves, the results of a subgroup including 12 patients who had received ranibizumab one month prior to the examination were analysed separately and no significant differences were observed in any of the study parameters between the ranibizumab-injected eyes and fellow eyes (The average values of the parameters for the ranibizumab-injected eyes and fellow eyes are as follows: 5.63 ± 0.57 vs. 5.75 ± 0.34 for the central corneal sensation, 24.48 ± 11.45 vs. 29.16 ± 10.44 for NFD, 33.33 ± 19.27 vs. 36.97 ± 11.95 for NBD, 16.49 ± 3.42 vs. 16.52 ± 4.41 for NFL, and 94.83 ± 6.60 vs. 89.92 ± 12.23 for the RNFL thickness; P>0.05 for all).

**Table 1:** Comparison of the study parameters in ranibizumab-injected eyes and fellow eyes of patients with neovascular age-related macular degeneration and the eyes of healthy control subjects.

	Ranibizumab-injected Eye (n = 33)	Fellow Eye (n = 33)	Control (n = 25)	Ranibizumab vs. Fellow	Ranibizumab vs. Control
				P value	P value
Central corneal sensation (mm)	5.72 ± 0.39	5.77 ± 0.31	5.82 ± 0.28	0.617^a^	0.336^b^
NFD (fibers/mm^2^)	27.46 ± 10.23	28.40 ± 9.12	29.74 ± 8.12	0.669^c^	0.362^d^
NBD (branches/mm^2^)	38.06 ± 17.70	40.52 ± 16.32	45.24 ± 19.45	0.482^c^	0.148^d^
NFL (mm/mm^2^)	16.30 ± 3.88	16.96 ± 3.95	17.45 ± 3.11	0.336^c^	0.231^d^
Average RNFL thickness (μm)	95.52 ± 10.10	93.55 ± 11.43	95.84 ± 13.75	0.237^c^	0.918^d^

^a^ Wilcoxon signed ranks test.

^b^ Mann-Whitney test.

^c^ Paired samples t-test.

^d^ Independent samples t-test.

Data are the mean ± SD. NBD, nerve branch density; NFD, nerve fiber density; NFL, nerve fiber length; RNFL, retinal nerve fiber layer.

Among the 33 patients with AMD, 11 (33.3%) had treated and controlled hypertension. There were no significant differences in any of the study parameters between patients with and without hypertension (P>0.05 for all).

No significant correlations were observed between the number of ranibizumab injections and any of the study parameters (P>0.05 for all). There was a significant positive correlation between central corneal sensitivity threshold and NFD (r = 0.362; P = 0.005).

## Discussion

The use of intravitreal injections of anti-VEGF agents for the treatment of retinal diseases, especially neovascular AMD, is now common practice. We found no significant change in corneal sensation or corneal nerve and retinal nerve fiber morphology in ranibizumab-injected eyes. This is reassuring given the theoretical concern of a potentially detrimental effect on nerves in the eye following anti-VEGF therapy, given that endogenous VEGF has neurotrophic and neuroprotective effects [20]. Indeed, Matsuzaki et al [21]. have demonstrated direct protective effects of VEGF-A on cultured neuronal cells. It has also been shown that a chronic decrease in endogenous VEGF-A levels is linked to an increased risk of motor neuron degeneration in amyotrophic lateral sclerosis [22]. In a more recent study, Hulse et al [23]. reported that VEGF-A165b, which is a splice isoform of VEGF-A, had neuroprotective and anti-nociceptive effects on epidermal sensory neurons in diabetic rats. In relation to neural changes there are experimental data showing the neuroprotective role of VEGF on retinal neural cells [8,24].

The role of VEGF on corneal innervation and nerve repair has also been investigated. Yu et al [10]. have shown a significant effect of VEGF on the growth of peripheral trigeminal neurons innervating the cornea in mice and suggested that blockage of VEGF signaling impairs regeneration of the corneal sub-basal nerve plexus after injury. Similarly, another recent study has demonstrated the neuroregenerative role of VEGF on corneal nerves using an avascular corneal nerve injury model [25]. These experiments provide support for the critical impact of VEGF on corneal and retinal neural cells and the potential for harm to ocular neural tissue in patients undergoing chronic anti-VEGF treatment. This provides the rationale for assessing the effect of ranibizumab on corneal nerves and peripapillary RNFL in patients with neovascular AMD.

Many studies have assessed the effects of anti-VEGF agents on the RNFL [11,13–15,26]. Theoretically, RNFL alterations could be induced by the IOP elevation in response to volume increase or through a direct neurotoxic effect of the drug. A short-term transient IOP rise occurs with intravitreal drug administration, but is limited to the first few minutes following injection and returns to baseline within 30–60 minutes [27,28]. We have not measured the IOP immediately after the injection and therefore cannot assess the contribution of this temporary rise in IOP to any effect on RNFL thickness. Although Martinez-de-la-Casa et al [11]. have demonstrated a significant decrease in RNFL thickness in 49 patients with neovascular AMD treated with a mean number of 4.8 ranibizumab injections, the majority of researchers have shown that repeated intravitreal injections of anti-VEGF agents are not associated with a change in RNFL [13–15,26]. Similarly in this study, we have also not observed any significant correlation between the number of injections and RNFL thickness.

Although several studies have investigated the effects of anti-VEGF on RNFL, no prior study has addressed the possible effects of intravitreal anti-VEGF agents on the corneal SBNP. Pharmacokinetic studies have shown that ranibizumab concentration peaks in aqueous humor 1–3 days after intravitreal injection and subsequently declines with a half life of 2.84 days in rabbits [29] and 7.19 days in human [30]. In an experimental study by Dratviman-Storobinsky et al [31], immunostaining showed bevacizumab in the anterior chamber and corneal stroma one day after intravitreal injection, suggesting that bevacizumab had reached the corneal stroma by passive diffusion via the endothelium. Since ranibizumab is a smaller molecule than bevacizumab, it is possible that ranibizumab may also reach the corneal stroma and have a deleterious effect on corneal nerves. To the best of our knowledge, this is the first study to investigate the morphology and function of corneal SBNP after repeated intravitreal injections of ranibizumab. We find no significant change in corneal sensation or corneal nerve morphology in ranibizumab-injected eyes. A limitation of this study is that the preinjection evaluations have not been performed due to the cross-sectional nature of the study. Further prospective studies with larger numbers of subjects including pseudophakic, aphakic, or vitrectomized eyes are recommended to fully evaluate the potential harmful effects of intravitreal injections of anti-VEGF agents.

In animal studies, the aqueous humor concentrations of ranibizumab in the non-injected fellow eye have found to be below the limit of detection and aqueous humor VEGF concentrations of the fellow eye remain unchanged following intravitreal injection [29,32]. Additionally, Gamulescu and Helbig [33] have reported no contralateral eye effect in a short-term prospective study of 26 patients with bilateral neovascular AMD who had received ranibizumab injection in one eye. Although Rouvas et al [34]. observed a positive impact of ranibizumab on the visual acuity of the fellow eye in patients with bilateral AMD. Given that a single intravitreal administration of 0.5 mg of ranibizumab in monkeys results in a maximum serum concentration of 150 ng/ml [35], a systemic effect on the contralateral eye should also be accounted for. Therefore a healthy control group was also included in the present study. No significant differences were observed between ranibizumab-injected eyes and the eyes of healthy controls for any of the parameters.

In conclusion, repeated treatment with intravitreal ranibizumab has no adverse effects on corneal sensation, corneal SBNP morphology and peripapillary RNFL thickness.
